# Does the Number of Harvested Sentinel Lymph Nodes Matter in Breast Cancer Patients with Limited Nodal Burden? A Descriptive Cohort Study

**DOI:** 10.3390/medsci14060689

**Published:** 2026-10-20

**Authors:** Geok Hoon Lim, Me Me Win Htein, Fuh Yong Wong, Maria Ileana Syliangco, Seyed Ehsan Saffari

**Affiliations:** 1 Breast Department, KK Women’s and Children’s Hospital, Singapore 229899, Singapore; me.me.w.h@singhealth.com.sg (M.M.W.H.); lianesyliangco@gmail.com (M.I.S.); 2 Surgery Academic Clinical Program, Duke-NUS Medical School, National University of Singapore, Singapore 169857, Singapore; 3 Department of Breast & Gynaecological Radiation Oncology, National Cancer Centre Singapore, Singapore 168583, Singapore; wong.fuh.yong@singhealth.com.sg; 4 Centre for Biomedical Data Science, Duke-NUS Medical School, National University of Singapore, Singapore 169857, Singapore; ehsan.saffari@duke-nus.edu.sg

**Keywords:** breast cancer, sentinel lymph node biopsy, axillary lymph node dissection, recurrence, axillary staging

## Abstract

**Background/Objectives**: In breast cancer patients with limited nodal burden, sentinel lymph node biopsy (SLNB) alone, without axillary lymph node dissection (ALND), has shown non-inferior survival. However, it remains unclear how many SLNs should be harvested. We aimed to determine if the number of harvested SLNs and the use of a single agent for SLNB localisation affect the detection of residual metastatic nodes. We also described the oncological outcome of SLNB alone as an exploratory clinical outcome. **Methods**: From 1 January 2020 to 31 January 2026, T1–2 patients and 1–2 metastatic SLNs who underwent SLNB only or with completion ALND were analysed. The number of SLNs harvested and the use of single or dual agents for localisation of SLNs were analysed in the ALND group to determine if it affected the detection of residual metastatic nodes. Data on oncological outcomes were reported. **Results**: A total of 152 patients were enrolled, of which 63 and 89 underwent SLNB alone and ALND respectively. The ALND group had more patients with mastectomy (*p* = 0.0044), macrometastasis (*p* = 0.0001) and no radiation to the axilla (*p* = 0.0001). Median number of harvested SLNs was 3 (range: 1–8) and 2 (range: 1–9) in the SLNB and ALND groups respectively. In the ALND group, a mean residual 2.4 metastatic nodes (range: 1–9) was seen in 24.7%. Multivariable logistic regression showed that a higher SLN yield was not associated with less residual disease (adjusted OR 0.71 per additional SLN, 95% CI [0.46, 1.12]). Conversely, the number of metastatic SLNs was statistically associated with residual nodal disease OR 3.6 95% CI [1.19, 10.86]. Single-agent SLNB localisation (mainly patent blue dye) was used in 22% and 19.3% of SLNB and ALND group respectively. Though dual-agent localisation had more patients with ≥2 SLNs (*p* = 0.0181), it was not statistically associated with residual nodal metastatic disease which occurred in 7/17 (41.2%) single-agent versus 15/71 (21.1%) dual-agent cases (*p* = 0.1184; OR 2.58, 95% CI [0.71, 9.09]). No recurrences were observed on a median follow-up of 37 (range: 3–75) months. **Conclusions**: The number of harvested SLNs and the use of patent blue dye as a single-agent localisation were not statistically associated with residual nodal disease in patients with limited nodal burden. Instead, metastatic SLN burden was statistically significant for residual nodal disease. However, our findings were limited by small sample size and should be considered as exploratory in nature with validation needed in larger studies with longer follow-up.

## 1. Introduction

In breast cancer patients with T1–2 disease and limited nodal burden of 1–2 metastatic lymph nodes on sentinel lymph node biopsy (SLNB), omission of axillary lymph node dissection (ALND) has shown non-inferior oncological outcomes [1]. While this was initially demonstrated in patients undergoing breast conserving surgery, this finding was also subsequently reported in patients undergoing mastectomy, T3 disease and in patients with extracapsular extension, albeit in smaller numbers [2,3,4].

While this practice has been adopted clinically in centres worldwide [5], it remains unclear what is the optimal number of SLNs to be harvested in cases with limited nodal burden. Conventionally, prior to Z11 trial, about two SLNs were harvested in patients with early breast cancer [6] and dual-agent localisation for SLNB was the gold standard [7].

Following Z11 trial, in the randomised controlled trials which demonstrated comparable survival between the groups with SLNB alone versus ALND [1,2,3,8,9,10,11], the median number of SLNs harvested in the SLNB alone group was also about two, with a range of one to eight lymph nodes. However, it remained unclear if there should be an optimal number of SLNs that should be harvested for accurate axillary staging in patients with limited nodal burden. This was especially the case when all the harvested lymph nodes showed metastatic disease, i.e., metastatic nodal burden of one out of total one SLN or two out of total two SLNs harvested respectively. In such cases when there is 100% complete involvement of the harvested SLNs, it is unknown if one should harvest additional SLNs or incidental non-SLNs to better stage the axilla, since these additional harvested lymph nodes could potentially alter management. However, harvesting more lymph nodes also means a higher risk of arm lymphedema and associated complications, hence this risk needs to be balanced against the clinical need for further axillary staging. Though there were such cases in the randomised controlled trials, they were small in numbers and there were no specific reported oncological outcomes for this group of patients.

In addition, the mode of SLNB localisation was not standardised in all the randomised controlled trials. While the use of dual agents for SLNB localisation could increase the yield of SLNs harvested, it is unknown if the use of a single agent could suffice in patients with limited nodal burden. This is of great clinical relevance since some countries with limited resources may only have access to a single agent for SLN localisation.

Hence, we aimed to determine if the number of harvested lymph nodes and the mode of SLNB localisation will affect the detection of residual nodal disease. As a secondary analysis, we also examined the association of complete SLN involvement with residual disease. As an exploratory clinical outcome, we described the oncological outcome of SLNB alone in patients with limited nodal burden.

This study has been reported in accordance with the STROCSS guidelines 2025 update [12].

## 2. Materials and Methods

Female breast cancer patients who were diagnosed with pathologically proven invasive T1–2 breast cancer and 1–2 metastatic SLNs at KK Women’s and Children’s Hospital, Singapore from 1 January 2020 to 31 January 2026 were recruited. Preoperatively, all patients were assessed to be clinically node-negative and had a radiologically negative ipsilateral axillary ultrasound.

Patients with neoadjuvant chemotherapy, synchronous or metachronous bilateral breast cancers, genetic mutation carriers [13], stage IV disease or who were lost to follow-up were excluded. Patients with T3/T4 breast cancer or had 3 or more metastatic SLNs on frozen section were also excluded.

Mastectomy with or without reconstruction or breast conserving surgery was determined by the patient’s tumour to breast size ratio, number of foci of cancer and preference. All patients underwent SLNB. The decision for ALND of level I and II during the operation was dependent on the surgeon after consideration of the individual patient’s clinical findings. During the operation, SLNB localisation can be done using dual or single agents based on surgeon’s preference. The common modes of localisation were radioisotope and patent blue dye. The number of lymph nodes harvested was based on intraoperative findings of hot or/and palpable or/and blue nodes. In this study, an incidental non-SLN node was defined as a node which was not detected using the modes of localisation during SLNB. The harvested nodes were then assessed intraoperatively using frozen section for metastatic disease. Isolated tumour cells (smaller than 0.2 mm of tumour deposit) in the lymph node were considered as node-negative. Micrometastasis and macrometastasis in the lymph nodes were defined as tumour deposits measuring between 0.2 mm to 2 mm and >2 mm respectively.

Patients who underwent SLNB only from February 2024, after approval of IRB for prospective study, were recruited prospectively whereas the remaining patients prior to this period and patients who underwent ALND were analysed using data from a prospectively kept database. All patients underwent metastatic staging with computed tomography and bone scan, and all had no evidence of distant metastasis. After surgery, they were discussed in a multidisciplinary board meeting and treatment was administered accordingly. Essentially, patients who had oestrogen receptor and/or Human epidermal growth factor receptor 2 (HER2) positivity were treated with hormonal therapy and/or HER2 targeted therapy respectively. Radiotherapy was usually given to patients who underwent breast conserving surgery. Nodal radiotherapy was defined as the receipt of axilla or/and supraclavicular/internal mammary lymph nodes radiation. The patients were then followed up at regular intervals with clinical examination and a yearly mammogram with supplementary breast ultrasound if clinically indicated.

Data collected included patient’s demographics, pathological features and oncological outcomes. Specifically, the number of lymph nodes which were harvested during SLNB and the mode of localisation were collected. This was analysed in the ALND group to determine their effect on the detection of residual nodal disease. Oncological outcomes were collected and reported as a secondary outcome. For this study, the follow-up period was calculated from the date of surgery to the most recent date the patient was last reviewed before 15 July 2026.

SingHealth Centralised Institutional Review Board approval (CIRB Ref. 2023/2735, date of approval 3 February 2024 and CIRB Ref. 2019/2419, date of approval 16 May 2019) was obtained for this study. Informed consent was given by patients who were recruited prospectively whereas there was a waiver of consent for the remaining patients.

### Statistical Analysis

In this descriptive cohort study, no formal sample size or power calculation was performed. Categorical variables were summarised as frequencies and percentages, while continuous variables were summarised using means or medians and ranges, as appropriate. Comparisons between groups were performed using Fisher’s exact test for categorical variables and the independent-samples *t*-test or Mann–Whitney U test for continuous variables, as appropriate.

Among patients undergoing ALND, multivariable logistic regression was used to evaluate factors associated with residual non-SLN metastasis. The model included the total number of SLNs harvested, the number of metastatic SLNs, and the presence of macrometastasis versus micrometastasis. The number of covariates was limited given the relatively small number of events. Firth penalised logistic regression was additionally performed as a sensitivity analysis to assess the potential influence of sparse-data bias. Results were expressed as odds ratios (ORs) with 95% confidence intervals (CIs).

The analysis of patients with complete involvement of the harvested SLNs (1/1 or 2/2 metastatic SLNs) was considered secondary and exploratory. All statistical tests were two-sided, and *p*-values below 0.05 were considered statistically significant. Statistical analysis was done using R software (version 4.4). Given the exploratory nature of the study and the multiple comparisons performed, findings were interpreted cautiously.

This study was registered with ClinicalTrials.gov (identifier: NCT06259513) on 6 February 2024.

## 3. Results

**Figure 1:**
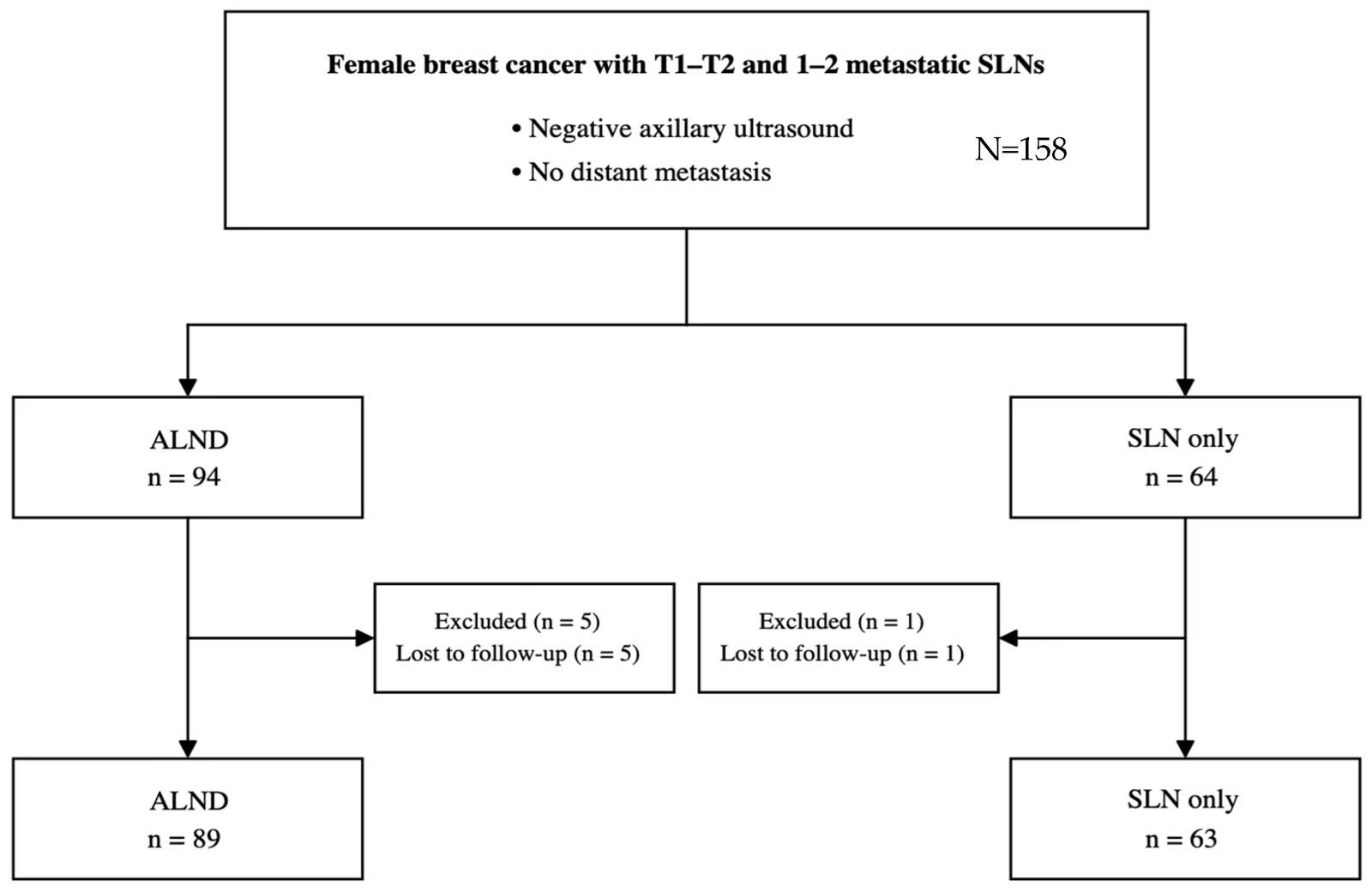
Flowchart of patient selection.

A total of 158 patients met the inclusion criteria. However, one and five patients from the SLNB only and ALND group were lost to follow-up (Figure 1). This resulted in 152 patients being analysed, of which 63 and 89 underwent SLNB alone and ALND respectively. The median age was 55 years old (range: 31–89) versus 56 years old (range: 34–95) and the mean tumour size was 24.7 mm (range: 7–45) versus 25.3 mm (range: 5–50) in the SLNB and ALND groups respectively. The ALND group had more patients with mastectomy (*p* = 0.0044), macrometastasis (*p* = 0.0001) and fewer patients who received radiation to the axilla (*p* = 0.0001) (Table 1).

The median number of harvested SLNs was three (range: 1–8) and two (range: 1–9) in the SLNB and ALND groups respectively. In the ALND group, an additional mean of 14.7 (range: 1–41) lymph nodes was harvested. From this group, 22/89 (24.7%) patients had residual nodal disease with a mean of 2.4 (range: 1–9) metastatic nodes on ALND, of which 3.4% had >3 metastatic lymph nodes. In the ALND group, residual disease was observed in 5/17 (29.4%) 95% CI [10.3, 56.0], 9/29 (31.0%) 95% CI [15.3, 50.8] and 8/43 (18.6%) 95% CI [8.4, 33.4], when one, two, or three or more SLNs were harvested. This absolute event rate did not rise with fewer harvested SLNs and in the adjusted model a higher SLN yield was not statistically associated with less residual disease (adjusted OR 0.71 per additional SLN, 95% CI [0.46, 1.12]) (Table 2). A likelihood-ratio test also confirmed that categorical SLN yield added no predictive information over metastatic burden (*p* = 0.249).

Macrometastasis was present in 39.7% and 91% of patients with SLNB only and ALND respectively (*p* = 0.0001). In subgroup analysis of the 81 patients with macrometastasis in the ALND group, higher SLN yield was not associated with residual disease (adjusted OR 0.72, 95% CI [0.46, 1.13]). The micrometastatic subgroup (N = 8) was too small for further analysis.

Though no statistically conclusive independent association between SLN yield and residual disease was demonstrated, the number of metastatic SLNs showed a significant association with residual nodal burden (adjusted OR 3.60, 95% CI [1.19, 10.86]).

In the Firth penalised multivariable logistic regression sensitivity analysis, the number of metastatic SLNs remained positively associated with residual non-SLN metastasis (OR 3.37, 95% CI [1.16–10.19]; *p* = 0.025). Similarly, no statistically significant associations were observed for the total number of harvested SLNs (OR 0.75, 95% CI [0.46–1.10]; *p* = 0.152) or the presence of macrometastasis (OR 1.33, 95% CI [0.25–13.53]; *p* = 0.761).

In view of the number of metastatic SLNs showing a significant association with residual nodal disease, we did a subgroup analysis of the 24 patients with exactly two metastatic SLNs to determine if the number of harvested lymph nodes would affect the detection of residual nodal disease. In this group of patients, residual nodal disease was found in 7/11 (63.6%) of those who harvested only two SLNs versus 3/13 (23.1%) of those who harvested more than two (*p* = 0.095; OR 5.37, 95% CI [0.75, 50.7]). With the metastatic burden held constant at two positive nodes, harvesting additional nodes was not associated with greater detection of residual disease. This is consistent with our principal finding that residual nodal burden is determined by the extent of SLN involvement rather than by the number of SLNs harvested. Given the small subgroup and wide confidence interval, we present this as an exploratory, hypothesis-generating analysis.

In the SLNB alone group, 14/63 (22%) were localised using a single agent. One patient had only radioisotope while 13 patients had only patent blue dye. From this group, 9/63 (14.3%) had a median of one non-SLNs harvested (range: 1–5), of which they were all negative for metastasis.

In the ALND group, of the 88 patients with data on localisation agents, 17/88 (19.3%) had single-agent localisation. Specifically, 2/88 (2.3%) and 15/88 (17%) had radioisotope and patent blue dye single-agent localisation respectively. Residual nodal disease occurred in 7/17 (41.2%) single-agent versus 15/71 (21.1%) dual-agent cases (Fisher’s exact *p* = 0.1184; OR 2.58, 95% CI [0.71, 9.09]). Given the small single-agent cell, we present this as exploratory and there was no statistically conclusive association demonstrated in this small subgroup. In the single-agent group, there were no reported technical difficulties or need for intraoperative conversion to ALND due to staging uncertainty. Though single-agent localisation retrieved fewer SLNs (median 2, mean 2.2, range 1–5) than dual-agent localisation (median 3, mean 2.8, range 1–9), this was not statistically significant (Mann–Whitney *p* = 0.14).

In the SLNB alone and ALND groups, 7/63 (11.1%) and 28/89 (31.5%) had 1/1 or 2/2 metastatic SLNs respectively. Of these patients, 2/7 (28.6%) and 27/28 (96%) in the SLNB and ALND group had macrometastasis respectively. Residual nodal metastasis occurred in 12/28 (42.9%) patients with complete SLN involvement versus 10/61 (16.4%) with partial involvement (*p* = 0.016); OR 3.76, 95% CI [1.23, 11.85]. The association persisted after adjustment for macrometastasis (adjusted OR 3.68, 95% CI [1.33, 10.18]) and within the macrometastatic subgroup (44.4% vs. 16.7%; OR 3.92, 95% CI [1.24, 12.96], *p* = 0.014). Given these findings, we attempted to determine if the number of harvested SLNs will affect residual nodal disease in these 28 patients with complete SLN involvement. In this subgroup analysis of 28 patients with complete involvement, residual nodal disease occurred in 5/17 (29.4%) of 1/1 cases and 7/11 (63.6%) of 2/2 cases (*p* = 0.12; OR 3.97, 95% CI [0.66, 28.6]). The residual-disease risk within the complete-involvement group therefore appears concentrated among 2/2 patients, again pointing to metastatic burden rather than harvest number as the determinant. As the subgroup is small and the comparison is confounded by the number of positive nodes, we interpret this cautiously as exploratory.

On mean follow-up of 30 months (range: 7–70) for these patients in SLNB alone group, no recurrence was observed. The recurrence rate in SLNB alone group and subgroup with complete SLN involvement with no ALND were 0/63, 95% CI [0, 5.7] and 0/7, 95% CI [0, 41.0] respectively. For all the patients in the study, no recurrence or deaths were observed after a median follow-up of 37 (range: 3–75) months.

## 4. Discussion

In this study, dual-agent localisation increased the yield of SLNs overall however this was not statistically associated with improved detection of residual metastatic nodal disease. Similarly, the number of harvested SLNs did not statistically affect the detection rate of residual metastatic nodes. Instead, the number of metastatic SLNs showed a significant association with residual nodal disease. In addition, patients with complete SLN involvement had a statistically higher risk of residual nodal disease compared to patients with partial involvement of harvested SLNs. No recurrence was observed in the entire cohort on intermediate-term follow-up. Due to the small sample size, the findings were best interpreted as exploratory.

ALND carries a high risk of arm co-morbidities such as shoulder dysfunction, arm lymphedema etc. which could affect the quality of life [8,14], hence ALND should be avoided where clinically possible. SLNB carries a low risk of arm lymphedema reported at 0–8% [15]. Increasing the number of SLNs harvested could improve the accuracy of axillary staging, as demonstrated in the National Surgical Adjuvant Breast and Bowel Project trial B-32 (NSABP B-32) study where the false negative rate was <10% for ≥2 LN, 6.9% for three SLNs, 5.5% for four SLNs and 1% for five or more SLNs [16]. However, harvesting more SLNs could also increase the risk of arm lymphedema. In fact, it was recommended that no more than five lymph nodes should be harvested during SLNB [17] as this could increase the risk of arm lymphedema. As a result, it is important to balance the accuracy of axillary staging with its potential complications.

After the publication of Z11 trial, there has been a decrease in the utilisation of ALND. Z11 trial findings were extrapolated to T1–2 breast cancer with clinically no nodal involvement regardless of molecular subtypes. This includes subtypes which are known to be more aggressive such as the triple negative and HER2 positive subtypes [18,19]. While studies showed no influence of the Z11 trial findings on the number of SLNs harvested [20,21], the number of SLNs to be harvested in these patients with limited nodal burden is poorly studied, with some proceeding to perform an ALND when only one SLN was harvested and it showed metastasis [22]. This was due to concerns that when there was complete involvement of the harvested SLNs, i.e., 1/1 or 2/2, this could be associated with a higher incidence of non-SLNs metastasis [23] and residual nodal disease could be present if SLNB alone was performed. Similarly, our study demonstrated that complete involvement of the SLNs were statistically significant for residual nodal disease. However, subgroup analysis of this group of patients showed that metastatic burden of the SLNs rather than the harvested number of SLNs was the determinant of residual nodal disease. This was similarly demonstrated in another paper which showed that two positive SLNs were associated with a higher risk of residual nodal disease than one positive SLN [24].

Though the incidence of non-SLNs metastasis could be higher in patients with higher SLN metastatic burden, it is unclear if this will translate into inferior oncological outcomes. In the Z11 trial, 27.3% of the patients had non-SLN metastasis on ALND. However, this did not translate to an inferior survival for the group who underwent SLNB only. This was likely because most of the patients in the trial received adjuvant therapy. Similarly, our study also had a comparable number of patients with non-SLN metastasis on ALND and most of our patients also received adjuvant treatment. Despite this residual nodal burden in our ALND group, there was no recurrence observed in our entire cohort. This was consistent with the results from another study which demonstrated that omission of ALND in patients with less than three SLNs did not increase the risk of disease recurrence [25].

The ideal mode of localisation of SLNs in patients with limited nodal burden is unclear. Conventionally, dual-agent localisation was reported to have a higher rate of identification of SLNs, often more than 98% with false negative rates below 5% compared to a single agent [26]. When used as a single agent, radioisotope tracer has a higher detection rate of around 95–98% and lower false negative rate than blue dye [27]. Conversely, blue dye alone has a detection rate of 85–95% and a false negative rate of 5% to 10% but this did not affect clinical outcomes [28]. As a result, in places with limited resources [29], blue dye, being a cheaper option, can be valuable for performing SLNB and reduce the rates of ALND and its complications. In our study, using patent blue dye as the single localisation agent did not statistically affect the detection of residual metastatic nodal disease. However, another study [30] reported that single-agent localisation with methylene blue dye could underestimate the number of positive SLNs compared to dual-agent localisation of methylene blue and indocyanine green fluorescence in patients with limited nodal burden. This finding will require further validation in future studies comparing the use of various localising agents including newer agents such as indocyanine green fluorescence and superparamagnetic iron oxide (SPIO) nanoparticles (Sentimag tracer) [31]. These newer agents could avoid the disadvantages of radiation and the small risk of anaphylaxis which are associated with radioisotope and blue dye respectively.

Besides SLNs, non-SLNs may be harvested as well during the operation due to intraoperative palpability, rather than being identified by the localising agents. While this may increase the yield of total harvested lymph nodes and decrease the false negative rates, the need to harvest these additional non-SLNs in patients with limited nodal burden is unclear. In our study, harvesting these non-SLNBs did not alter the management hence it may not be necessary to harvest them, though this finding was based on small numbers and hence should be interpreted with caution.

While the oncological safety of omitting ALND in patients with limited nodal burden has been reported in patients with breast conservation or micrometastasis, there is less data on this in patients with mastectomy and macrometastasis. Recent evidence from the SINODAR-ONE trial and the SENOMAC trial showed non-inferior oncological outcomes for mastectomy patients with limited nodal burden without ALND [32]. While these are promising results [2,3,33], further studies, such as the ongoing POSNOC trial, are needed to validate these findings to refine our management of this group of patients. This could explain why our study had statistically more patients with mastectomy, macrometastasis and no radiation to the axilla in the ALND group. This was likely due to the cautious adoption of omission of ALND in mastectomy patients with macrometastasis in view of current limited data in this area. As a result, these substantial confounding factors might affect the direct comparison of oncological outcomes and residual nodal burden between the two groups, hence results should be interpreted with caution.

Finally, in this era when the management of breast cancer is based largely on molecular markers such as ER, PR and HER2, there is less reliance on axillary staging for guidance of treatment. In patients with ER positive, HER2 negative and N1 disease, genomic testing could be used to determine the need for chemotherapy, as demonstrated in the RxPONDER (A Clinical Trial RX for Positive Node, Endocrine Responsive Breast Cancer) study [34]. In addition, while more than three metastatic lymph nodes were previously required in ER positive, HER2 negative breast cancer to start adjuvant CDK 4/6, this has changed with the findings from Natalee trial [35]. This latter trial demonstrated a reduction of cancer recurrence risk by 28.5% and included patients with N1 disease and unfavourable characteristics [36]. As a result, ALND need not be performed for staging purposes to guide treatment. In our study, only 3.4% had N2 disease which could have changed management.

This study has some strengths. Its strengths included that for prospectively enrolled patients who had SLNB alone, few were lost to follow-up. This is also one of the few studies which investigated the relationship of the number of harvested lymph nodes to residual nodal disease. This has practical implications and could potentially provide more clarity on the number of SLNs to be harvested during the operation. In addition, all patients had a negative axillary ultrasound preoperatively. Hence, this study is relevant in real-world settings to determine the number of lymph nodes which need to be harvested in patients with nodal burden not detected radiologically as more centres adopt ultrasound imaging of the axilla following SOUND [37] and INSEMA trial findings [38]. However, these trials were not done in Asian women, hence we eagerly await the results in an ongoing Asian study [39].

However, the study was not without limitations, which included a small sample size from a single centre and an intermediate-term follow-up. Particularly, our study had a small number of patients with total involvement of the harvested lymph nodes, i.e., 1/1 or 2/2 metastatic SLNs which were treated by SLNB alone. Though this group had no recurrence on intermediate-term follow-up, this promising result still needs to be validated in larger studies with longer follow-up. An event-free recurrence endpoint also provided very limited statistical information. A longer follow-up was also needed to validate our results since patients with ER positivity tend to recur late and this group of patients made up >90% of our cohort. In addition, as no formal sample size calculation was performed, and due to the small sample size of patients in certain subgroups, which limits further statistical analysis, the findings in this study were best considered exploratory but could provide insights for future studies. Moreover, in our study, the single-agent group comprised mainly of patients with patent blue dye, hence our results could only be extrapolated to the use of patent blue dye and cannot be generalised to all single-agent strategies. The other caveat was that only patients with successfully identified metastatic SLNs entered the cohort, hence the overall identification rate and false negative rate cannot be estimated. In our study, there were also statistically more patients with mastectomy, macrometastasis and less axillary radiation in the ALND group. This was likely secondary to clinician’s selection bias since there is currently limited evidence on the safety of omission of ALND in this group of patients. This, together with the mixed prospective/database-derived design of our study, makes the two groups not directly comparable; hence, caution should be exercised when interpreting the results. 

## 5. Conclusions

In conclusion, the number of harvested SLNs and the use of patent blue dye as a single agent for localisation were not statistically associated with residual nodal disease. Instead, SLN metastatic burden showed a significant association with residual nodal disease on ALND. No recurrence was observed in the cohort on intermediate-term follow-up. Due to a small sample size and relatively short follow-up, our findings should be considered as exploratory in nature with validation needed in larger studies with longer follow-up.

## Figures and Tables

**Table 1:** Characteristics of patients who underwent sentinel lymph node biopsy (SLNB) alone versus axillary lymph node dissection (ALND).

Clinical Features	No. (%) of Patients with SLNB Alone N = 63	No. (%) of Patients with ALND N = 89	*p*-Value
Age (years)			0.8672
≤50	26 (41.3)	35 (39.3)	
>50	37 (58.7)	54 (60.7)	
Pathological features			
Breast tumour histology			0.4910
Invasive ductal cancer	49 (77.8)	68 (76.4)	
Invasive lobular cancer	5 (7.9)	12 (13.5)	
Others	9 (14.3)	9 (10.1)	
Grade			0.2425
I	4 (6.4)	11 (12.3)	
II	47 (74.6)	55 (61.9)	
III	12 (19.0)	23 (25.8)	
Oestrogen receptor (ER)			0.7418
Positive	58 (92.1)	84 (94.4)	
Negative	5 (7.9)	5 (5.6)	
Progesterone receptor (PR)			0.1148
Positive	57 (90.5)	72 (80.9)	
Negative	6 (9.5)	17 (19.1)	
Human epidermal growth factor receptor 2 (HER2)			0.8220
Positive	9 (14.3)	15 (16.9)	
Negative	54 (85.7)	74 (83.1)	
pT #			1.0000
T1	27 (42.9)	37 (41.6)	
T2	36 (57.1)	52 (58.4)	
Number of SLN harvested			0.2007
1	6 (9.5)	17 (19.1)	
2	19 (30.2)	29 (32.6)	
>2	38 (60.3)	43 (48.3)	
Number of metastatic lymph nodes on ALND			NA
0	NA	67 (75.2)	
1	NA	11 (12.4)	
>1	NA	11 (12.4)	
Type of lymph node metastasis			0.0001
Micrometastasis	38 (60.3)	8 (9.0)	
Macrometastasis	25 (39.7)	81 (91.0)	
Surgical management of the breast			0.0044
Mastectomy	30 (47.6)	63 (70.8)	
Breast conserving surgery	33 (52.4)	26 (29.2)	
Adjuvant radiotherapy			0.0760
Yes	55 (90.2)	70 (78.7)	
No	6 (9.8)	19 (21.3)	
Missing	2	0	
Chest wall radiation after mastectomy (N = 93)	N= 30	N = 63	0.4715
Yes	22 (75.9)	42 (67.7)	
No	7 (24.1)	20 (32.3)	
Missing	1	1	
Breast radiation after breast conserving surgery (N = 59)	N = 33	N = 26	0.4987
Yes	30 (93.7)	25 (100)	
No	2 (6.3)	0	
Missing	1	1	
Axilla radiation			0.0001
Yes	28 (46)	3 (3.4)	
No	33 (54)	84 (96.6)	
Missing *	2	2	
Supraclavicular node radiation			0.2262
Yes	35 (57.4)	59 (67.8)	
No	26 (42.6)	28 (32.2)	
Missing *	2	2	
Internal mammary node radiation			0.5253
Yes	3 (4.9)	7 (8)	
No	58 (95.1)	80 (92)	
Missing *	2	2	
Adjuvant chemotherapy			1.000
Yes	42 (66.7)	59 (66.3)	
No	21 (33.3)	30 (33.7)	
Adjuvant Her2 therapy			1.000
Yes	8(12.7)	11 (12.4)	
No	55 (87.3)	78 (87.6)	
Adjuvant Endocrine therapy			0.8146
Yes	55 (87.3)	76 (85.4)	
No	8 (12.7)	13 (14.6)	
Recurrence			Exact 95% CI reported in text
Yes	0 (0)	0 (0)	
No	63 (100.0)	89 (100.0)	

# If multifocal/centric disease was present, the breast tumour size measurement will be based on the largest lesion. * Missing values due to patients having radiotherapy at another centre, hence details not available.

**Table 2:** Multivariable logistic regression for residual nodal metastasis in the ALND cohort (N = 89, 22 events).

Variable	Adjusted OR	95% CI
Number of harvested SLNs (per node)	0.71	0.46–1.12
Number of metastatic SLNs	3.60	1.19–10.86
Macrometastasis (vs. micrometastasis)	1.77	0.20–16.02

Odds ratios estimated by logistic regression; a likelihood-ratio test showed that categorical SLN yield (1, 2, ≥3) added no predictive information over metastatic burden (*p* = 0.249) and a Firth penalised model gave concordant estimates.

## Data Availability

The original contributions presented in this study are included in the article. Further inquiries can be directed to the corresponding author.
